# Sensitive Spectroscopy of Acetone Using a Widely Tunable External-Cavity Quantum Cascade Laser

**DOI:** 10.3390/s18072050

**Published:** 2018-06-27

**Authors:** Faisal Nadeem, Julien Mandon, Amir Khodabakhsh, Simona M. Cristescu, Frans J. M. Harren

**Affiliations:** Trace Gas Research Facility, Molecular, and Laser Physics, Institute for Molecules and Materials, Radboud University, Heyendaalseweg 135, 6525 AJ Nijmegen, The Netherlands; julien.mandon@gmail.com (J.M.); A.khodabakhsh@science.ru.nl (A.K.); S.cristescu@science.ru.nl (S.M.C.); F.harren@science.ru.nl (F.J.M.H.)

**Keywords:** laser sensors, infrared spectroscopy, semiconductor lasers, quantum cascade lasers, wavelength modulation spectroscopy, optical instruments

## Abstract

We employed a single-mode, widely tunable (~300 cm^−1^) external-cavity quantum cascade laser operating around 8 µm for broadband direct absorption spectroscopy and wavelength modulation spectroscopy where a modulation frequency of 50 kHz was employed with high modulation amplitudes of up to 10 GHz. Using a compact multipass cell, we measured the entire molecular absorption band of acetone at ~7.4 µm with a spectral resolution of ~1 cm^−1^. In addition, to demonstrate the high modulation dynamic range of the laser, we performed direct absorption (DAS) and second harmonic wavelength modulation spectroscopy (WMS-2f) of the Q-branch peak of acetone molecular absorption band (HWHM ~10 GHz) near 1365 cm^−1^. With WMS-2f, a minimum detection limit of 15 ppbv in less than 10 s is achieved, which yields a noise equivalent absorption sensitivity of 1.9 × 10^−8^ cm^−1^ Hz^−1/2^.

## 1. Introduction

In laser-based absorption spectroscopy, ultrasensitive absorption measurements are of particular interest for various applications such as environmental monitoring [1,2] and medical breath analysis [3,4,5,6]. One way to achieve highly sensitive measurements is to measure the absorption of the molecular species in the mid-infrared wavelength region, where most of them have their strongest ro-vibrational molecular transitions. Sensitivities levels up to parts-per-billion (ppbv) and even parts-per-trillion (pptv) are achievable for molecules with isolated absorption lines, using tunable laser-based absorption spectroscopy [2,7,8]; however, achieving equivalent sensitives for larger molecules with broadband absorption features is still a challenging task.

For trace gas sensing, single mode laser operation is highly desirable, along with a broad tuning range. Compact and reliable mid-infrared (mid-IR) sources for spectroscopic sensing mostly include quantum cascade lasers (QCLs) [9] or interband cascade lasers (ICL) [10]. These lasers achieve single mode operation by placing a distributed feedback (DFB) structure on top of the active gain medium. Single DFB-lasers show a high performance, but their tuning range is limited to a few wavenumbers. The tuning is performed either by current injection or by temperature tuning of the laser chip. Tuning by injection current is fast, with a maximum tunability range of ~5 cm^−1^ [11], while temperature tuning is slow with a tuning coefficient of 0.1–0.3 cm^−1^/K [12]. However, the disadvantage of both tuning mechanisms is the induced variation in the output power of the DFB lasers by changing the injection current (direct effect) or temperature (indirectly due to induced thermal effects on the gain medium) [12].

Contrary to DFB-based lasers, external cavity QCL (EC-QCL) provides broadband tuning (~300 cm^−1^) with high power (~200 mW), narrow line width, single mode, and mode-hop free operation over a limited tuning range [13,14,15,16,17]. EC-QCL can operate either in a narrowband or broadband scanning mode. In the narrowband scanning mode, it acts like a DFB laser and can target the individual molecular absorption lines, over a sweep of 2–5 cm^−1^ at a subsecond time scale [9]. Alternatively, to target molecules with broadband absorption features, broadband tuning (>250 cm^−1^) is achieved by rotating the diffraction grating of the external cavity. This enables identification of the individual molecular absorption lines and dense spectral structures of larger molecules in a few seconds. Due to their broadband tuning and flexibility in wavelength selection, EC-QCLs can be tuned precisely, but also swept quickly to enable multispecies detection, while skipping spectral regions with strong absorption from interfering species such as carbon dioxide (CO_2_) and water (H_2_O) in the atmosphere.

Standard Fourier transform infrared spectroscopy (FTIR) is considered as a benchmark due to its broadband spectral coverage spanning over the entire mid-infrared spectral region and highly accurate wavelength scale. In addition, the spectra can be averaged for a long time to reach high detection sensitivities [18]. However, FTIR systems are bulky, expensive, have a short interaction path length and require a long acquisition/averaging time to achieve high sensitivity. In comparison to the FTIR, spectroscopic techniques based on EC-QCLs are low cost, compact, and offer longer interaction path length for field measurements. The tuning range of EC-QCLs is smaller compared to the FTIR, but it is still sufficient to acquire broadband spectra of different larger species (e.g., acetone or ethanol) as well as the fine spectral structure of lighter species (e.g., N_2_O or HCN) both quickly and accurately. Since EC-QCLs are the only low cost and highly tunable laser source in the mid-IR, they are widely used in combination with various detection methods to exploit the strongest absorption lines of different molecules [19,20].

EC-QCLs, however, suffer from mechanical instability of the external cavity due to optomechanical tuning. Specifically, rotation of the grating in the external cavity introduces mode-hops due to the change of the laser cavity length and results in a spectral mismatch in the recordings of consecutive scans. Consequently, in the broadband tuning of the EC-QCL, low noise levels are hard to achieve due to the difficulty of averaging the consecutive scans efficiently [18]. This effect is reduced significantly in the narrowband scan which is performed by tuning the piezo actuator(s) mounted on the diffraction grating. The mechanical instability issue in the broadband-scanning operation of the EC-QCL can also be mostly overcome by precise repositioning of the external length of the cavity. For instance, a mode tracking system can be implemented to adjust the length of the EC-QCL cavity and the angle of the grating simultaneously, enabling an active control of the wavelength in the EC-QCLs. Consequently, wide scan operation over a full gain bandwidth of the QCL can be made vibration-free with equivalent precision to a typical DFB-QCL [13].

Several spectroscopic methods are available to detect the concentration of gas samples with high sensitivity; among those direct absorption spectroscopy (DAS) is the easiest and the most common. To achieve high sensitivity a long absorption path length is needed, which can be obtained using multipath cells, such as White [21], Herriott [22] or astigmatic cells [23] up to few hundred meters. To reach kilometer-long optical path lengths, cavity-enhanced methods such as cavity ring-down spectroscopy (CRDS) [24] and off-axis integrated cavity output spectroscopy (OA-ICOS) [8,25,26] are utilized. Although these methods provide a long optical path length, coupling the laser light into a high-finesse cavity is challenging. For best performance, CRDS requires laser stabilization schemes [27] which is not needed for OA-ICOS, however the performance of OA-ICOS remains limited by the low laser power throughput [28].

Wavelength modulation spectroscopy (WMS) with higher harmonic detection is often used as an alternative way to effectively reduce the source and electronic 1/f noise in the system. By normalizing the wavelength modulation second harmonic (2f) signal on the first harmonic (1f) signal, it also enables a calibration-free approach [29]. WMS can be extended to large modulation amplitudes using EC-QCLs because these sources can be rapidly tuned via large modulation currents [30]. In this regard, the laser frequency is modulated using a rapid current modulation to maximum achievable modulation amplitude, while it is also scanned slowly via a piezo actuator (PZT) across the absorption transition of the molecule. This technique is often called scanned WMS.

DFB-QCLs can also achieve rather wide tuning ranges up to 20 cm^−1^ using integrated heaters [31,32]; however, the dynamic of this tuning is not fast enough (usually limited to a few kHz) to be used for modulating the wavelength of the laser for WMS. The heaters are generally used for scanning the wavelength of the laser over the absorption feature while wavelength modulation is achieved by modulating the current of the laser, the same process which is used in WMS with EC-QCLs. A number of earlier publications highlighted EC-QCL-based scanned WMS. Briefly, we review the relevant aspects of WMS in terms of sensitivity compared to DAS. Rao et al. demonstrated WMS with the EC-QCL for the first time achieving a 16-times better sensitivity compared to DAS, for the detection of nitrogen dioxide (NO_2_) near 1632.51 cm^−1^ [33]. Li et al. showed the wavelength modulation spectroscopy with a modulation amplitude of ~20 GHz using EC-QCL for the detection of water vapors in the spectral region spanning from 7201–7206 cm^−1^ [34]. Other researchers followed for the detection of other gases using single or multiple molecular absorption lines [20,35,36,37]. However, achieving high sensitives for larger molecules via scanned WMS is still remain a demanding task [38].

In this work, we combine a high power, single-mode, widely tunable EC-QCL centered at 8 µm, with a 76 m multipass cell for scanned DAS and WMS-2f. We target the peak in the Q-branch absorption band of acetone (Half width Half Maximum (HWHM) ~10 GHz) around 1365 cm^−1^ with a high modulation amplitude to provide a frequency excursion of around 10 GHz. Scanned broadband DAS and narrowband DAS signals are compared to the data from the Pacific Northwest National Laboratory (PNNL) database, while WMS-2f data are fitted by a developed analytical model. A comparison between scanned DAS and WMS-2f is further elaborated. The system presents a combination of high sensitivity, resolution, and acquisition speed using a single QCL with continuous tuning control over the central emission wavelength.

## 2. Definitions of Wavelength Modulation Spectroscopy (WMS)

In frequency modulation spectroscopy (FMS) the modulation frequency is in the RF domain (the same order of magnitude of the width of the measured absorption line) and the modulation amplitude is very small; in WMS the modulation frequency is small (usually in the tens of kilohertz range), while the modulation amplitude is much larger (in the RF domain and close to the absorption linewidth). In WMS, when we apply a sinusoidal modulation with a frequency of $\omega_{m}$ and depth of β to the laser injection current, the electric field can be written as [39],

(1) $$E\left(t\right)=\frac{E_{0}}{2}e^{i\left[\omega_{c}t+\beta \sin\left(\omega_{m}t\right)\right]},$$

where $\omega_{c}$ is the carrier frequency. This yields an instantaneous laser frequency of $\omega_{i}$ which can be calculated as the derivative of the phase [40,41],

(2) $$\omega_{i}\left(t\right)=\omega_{c}+\beta \omega_{m}\cos\;(\omega_{m}t),$$

where $\Delta \omega_{L}=\beta \omega_{m}$ is the modulation amplitude and equal to the product of the modulation depth (*β*) and modulation frequency $\left(\omega_{m}\right)$. Using WMS, with a modulation amplitude close to 2.2 times of Γ, the HWHM of the absorption linewidth of interest, provides a sensitivity comparable to FMS [40]. Although, FMS offers advantages of suppressing the laser excess noise and etalon effects, it also complicates the system. For instance, one challenge to implement FMS is the performance of the lock-in amplifier which is limited for modulation frequencies. In WMS, for atmospheric pressure broadened absorption lines, the modulation amplitude $\Delta \omega_{L}$ will be in GHz range, while modulation frequency $\omega_{m}$ is in the kHz range [42]. For instance, for *β* = 10^5^ and $\omega_{m}=50$ kHz the modulation amplitude is 5 GHz. The detector signal is demodulated using a lock-in amplifier, where it is multiplied with the reference signal (modulation source) at the n^th^ component of the modulation frequency and is further passed through a low pass filter. It is usual practice to measure the absorption profile at the 2nd harmonic (WMS-2f) as the 1st harmonic (WMS-1f) is affected by the linear background [42,43].

## 3. Simulation of Absorption Lines

**Figure 1:**
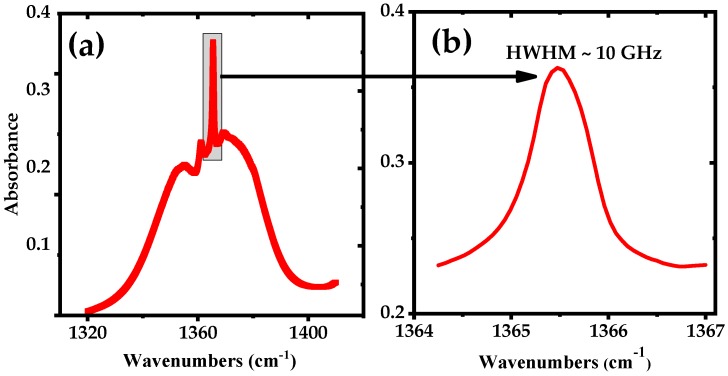
Simulated spectrum using the PNNL 2014 database, for 50 ppmv mixture of acetone in nitrogen (effective path length of 76 m, pressure 100 mbar and temperature 298K). (**a**) Broadband absorption spectrum of acetone and (**b**) narrowband spectrum of the Q-branch peak of acetone at 1365 cm^−1^.

Figure 1a shows the calculated absorption spectrum of a mixture of 50 ppmv of acetone in N_2_, for an absorption path length of 76 m and a pressure of 100 mbar at room temperature, derived from the PNNL database [44]. The Q-branch peak of the acetone spectrum centered at 1365.49 cm^−1^ is enlarged in Figure 1b and has a HWHM of Γ ~ 10 GHz.

The theory and modelling of WMS is extensively investigated in the literature; it has been shown that by increasing the modulation amplitude up to $\;\Delta \omega_{L}\approx 2.2\Gamma$, the magnitude of the WMS-2f line shape and hence the signal-to-noise ratio (SNR) increases. However, by further increasing the modulation amplitude, the WMS-2f signal begins to decrease and finally it splits into two separate parts. Therefore, the best ratio of the modulation amplitude and absorption linewidth to achieve the highest SNR is around 2.2 [35]. According to this, to scan Q-branch peak of acetone (10 GHz), a modulation amplitude of 22 GHz is required to maximize the WMS-2f signals for optimum SNR. The maximum modulation amplitude of our EC-QCL is ~10 GHz, limited by the dynamic range of the injection current modulation. We use this value instead of 22 GHz, which limits the maximum signal to ~60% of the maximum achievable WMS-2f signal.

## 4. Experimental Set-Up

**Figure 2:**
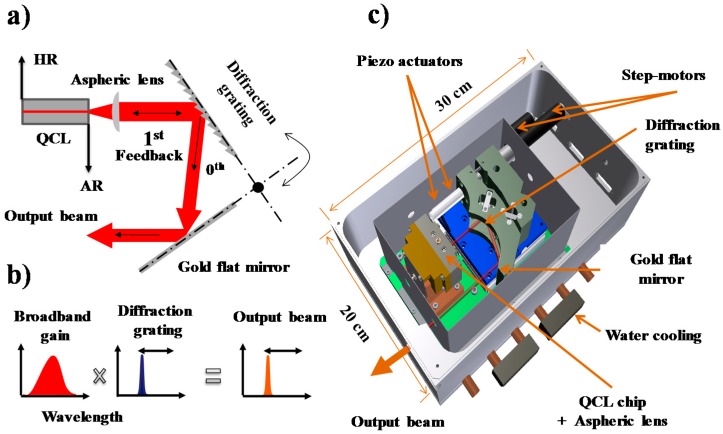
(**a**) A sketch of the Littrow configuration used in the EC-QCL design; (**b**) controlling the emission wavelength of the broadband gain QCL chip by the diffraction grating; (**c**) three-dimensional design of the laser housing, where thermoelectrically cooled Fabry–Pérot QCL gain chip is mounted vertically on a copper block together with three-dimensional adjustable aspheric lens.

In our experiments, we used a homemade EC-QCL with a specially designed laser housing. Since a detailed laser layout and operation was described previously [15], here we briefly summarize the key parts. A Fabry–Pérot QC laser chip (Alpes Lasers, Saint-Blaise, Switzerland) was used as the gain medium. The chip was HR coated at rear facet and AR coated at the front facet and it was attached to a copper block with a homemade XYZ-translational stage. The copper block was cooled with an additional stage thermoelectric cooler to provide long-term, low-temperature (−30 °C) stability to the chip during the laser operation. A laser diode driver (LDX-3232, ILX Lightwave, Newport, Bozeman, MT, USA) was used to provide the current to the laser chip, with a noise level of 20 µA in low bandwidth operation. The external cavity was designed in a *Littrow* configuration shown in Figure 2a consisting the gain chip, an aspherical collimating lens (focal length 3 mm, AR coated for 8–12 µm, Light Path, Orlando, FL, USA), and a diffraction grating. The diffraction grating was gold coated and blazed at 10.6 µm (150 grooves/mm, Optometrics, Ayer, MA, USA). The 0th order reflection of the grating was used for out-coupling. A flat, gold-coated mirror, positioned under 90 degrees for a cat’s eye reflection, reflects the out-coupled light. The wavelength selection mechanism of the laser is shown in Figure 2b. In this approach, the laser beam was neither displaced nor rotated during wavelength scanning. A complete sketch of the system is shown in Figure 2c. The diffraction grating and mirror were placed on a translation/rotation stage, which allowed to change the cavity length and rotate the grating angle, independently. A DC motor (M-227.10, Physik Instrumente, Karlsruhe, Germany) controlled the coarse and fine-tuning of the grating. Wavelength scanning over a limited range was achieved with a piezo actuator (P-840.60, Physik Instrumente, Karlsruhe, Germany, 100 Hz closed-loop bandwidth) mounted between the DC motor and the diffraction grating.

Furthermore, to adjust the laser cavity length another translation stage was used in combination with an additional piezo actuator, such that a maximum length change of up to 25 mm and a maximum angle change of up to 10 degrees was achieved. To perform real-time measurements, a LabVIEW program is developed to control all the drivers of the actuators and motors. Since the laser is operating at low temperature, water condensation can deposit ice on the QCL gain chip and optical elements of the laser cavity. Therefore, the enclosure was continuously flushed with a N_2_ flow (<1 Lh^−1^), especially the space between the laser gain chip and the collimation lens. The external cavity was placed in a stainless steel box (for radiation shielding), which was surrounded by another aluminum box (overall dimensions 30 × 20 × 15 cm). As the box was flushed with N_2_, the laser beam exits the housing via a ZnSe window (diameter 2.54 cm) placed at Brewster angle for the polarized EC-QCL laser beam.

**Figure 3:**
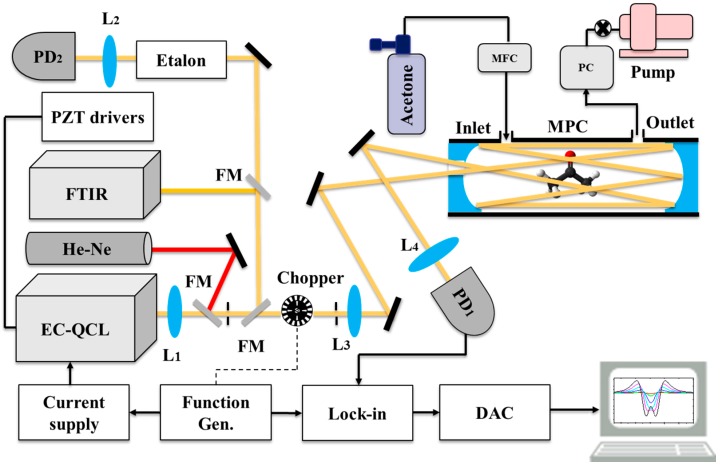
Experimental setup of the spectrometer using the home-built EC-QCL and a multipass cell. The modulation reference is generated by either a chopper (DAS) or function generator (WMS-2f). The output signal from the detector is sent to a lock-in amplifier to detect the direct or 2f signal. The output of the lock-in amplifier is digitized by a data acquisition card and analyzed in a PC. MPC: multipass cell, FM: flip mirror, PZTs: piezo drivers, L: lens, FTIR: Fourier transform infrared spectrometer (green lines), PD: photodetector, EC-QCL: external cavity quantum cascade laser, PC: pressure controller, MFC: mass flow controller. Black lines: BNC cables, dotted black lines: alternative connection, yellow lines: EC-QCL beam, red lines: He-Ne laser beam.

The EC-QCL was used in combination with a multipass cell (AMAC-76, Aerodyne Inc., Billerica, MA, USA) with an effective length of 76 m, as shown in Figure 3. The mirrors of the multipass cell (MPC) were coated for optimal reflection (reflectivity ~99.2%) between 3–12 µm. Two lenses L_1_ (f = 24 cm, ZnSe) and L_3_ (f = 35 cm, ZnSe) were used to match the waist of the beam to the middle of the MPC. The laser beam exiting the MPC was focused onto an infrared detector (PD_1_, PVI-4TE-10.6, Vigo Systems, Ożarów Mazowiecki, Poland). A lock-in amplifier (Stanford Research Systems, Sunnyvale, CA, USA) was used to demodulate the signal from the detector. The demodulated signal was recorded via a data acquisition card (NI PCI-6259, Austin, TX, USA) and a LabVIEW program for further analysis.

We verified the effective optical path length of the MPC by measuring a single absorption line of 5 ppmv N_2_O in N_2_ at 1300.92 cm^−1^ with a single pass cell and the MPC. By comparing the two measured absorption lines, we verified that an optical path length of 76 m is achieved in the MPC. A mass flow controller (Brooks Instruments, Hatfield, PA, USA) regulated the flow of the sample gas passing through the MPC. During the measurements, a constant flow rate of 3 Lh^−1^ was maintained through the MPC at a pressure of 100 mbar using a vacuum pump and a pressure controller. The sample gas was provided either by a calibrated bottle (Linde Gas, Dieren, The Netherlands) of 50 ppmv of acetone in N_2_ or by a pure N_2_ gas bottle for background measurement.

A Fourier Transform Infrared Spectrometer (FTIR, Nicolet Magna 560, Thermo Scientific, Waltham, MA, USA) as well as a germanium Etalon (FSR = 1.5 GHz) in combination with a focusing lens L2 (f = 3 cm, ZnSe) and an IR detector PD2 (liquid N_2_ cooled, Kolmar Technologies, Newburyport, MA, USA) were used for wavelength calibration. The wavelength drift of the broadband tuning of the EC-QCL was verified to be below 0.1 cm^−1^ (3 GHz) over a week period. A He-Ne laser in combination with two pinholes was used for optimal alignment of the infrared beam to the MPC. DAS measurements were performed by mechanically chopping the laser beam, while for WMS a function generator was used to modulate the injection current to the EC-QCL by a sinewave.

## 5. Characterization of the EC-QCL

**Figure 4:**
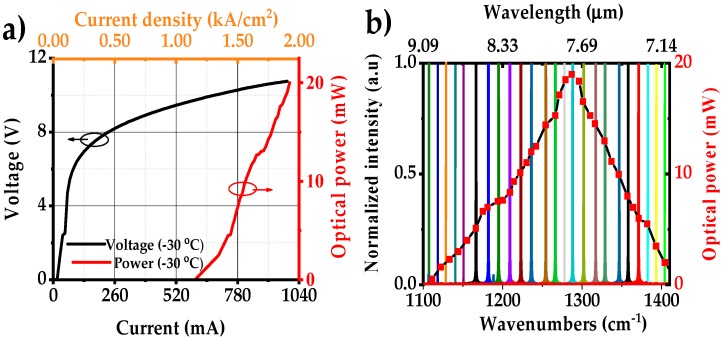
(**a**) L-I-V curves of the EC-QCL; (**b**) normalized FTIR spectral tuning coverage of EC-QCL with corresponding output powers at −30 °C. The total scanning range is over 300 cm^−1^ with a maximum output power of 20 mW.

Light–current–voltage (L-I-V) characteristics of the external cavity laser are shown in Figure 4a. The laser operated up to 990 mA (safety limit 1.0 A) with a maximum output power of 20 mW (T = −30 °C). The tuning range of the EC-QCL and the output power are shown in Figure 4b. The tuning range was recorded by an FTIR with a resolution of 0.125 cm^−1^. The EC-QCL has a maximum scanning range of 300 cm^−1^ centered around 1250 cm^−1^ (8 µm) providing single-mode operation over >250 cm^−1^. The output power of the laser is >10 mW over more than 100 cm^−1^. A complete tuning performance of the EC-QCL is shown in Table 1.

The frequency response of the laser is characterized by modulating the current on the laser chip and observing its frequency response through the etalon (FSR 1.5 GHz) by an infrared detector (PD_2_). A signal generator (33220A, Agilent, Santa Clara, CA, USA) providing a sinewave is used to modulate the laser through a laser diode current source (LDX-3232, ILX Lightwave, Newport, Bozeman, MT, USA). A reference signal from the function generator is sent to the lock-in amplifier to demodulate the signal.

From the response of the etalon around 1300 cm^−1^ at a maximum power of 20 mW, we estimated that a maximum modulation amplitude of ~10 GHz is achieved before reaching to 100% amplitude modulation of the injection current. For WMS-2f, we further optimized the experimental parameters (pressure, modulation frequency, and phase) to maximize the WMS-2f signal.

## 6. Direct Absorption and Wavelength Modulation Spectroscopy

**Figure 5:**
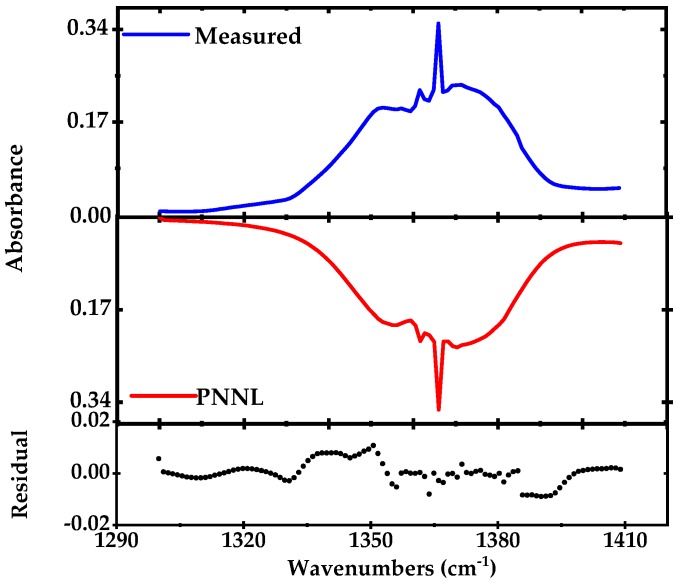
Direct absorption spectrum of a complete ro-vibrational band of acetone (1300–1420 cm^−1^) recorded in 10 s and averaged for five times in blue (100 mbar pressure, the optical path length of 76 m) compared to simulated absorption spectrum from the PNNL 2014 database in red [44]. The lower panel shows the residual after subtraction of the measured from the simulated profile.

For broadband absorption measurements, the EC-QCL output intensity is modulated with a mechanical chopper (frequency ~ 1 kHz) and demodulated using a lock-in amplifier at the output of the detector. We measure the entire vibrational band of acetone (50 ppmv in nitrogen) spanning over 100 cm^−1^ around 1360 cm^−1^, at 100 mbar pressure and room temperature, as shown in Figure 5. The spectrum is recorded by continuously tuning the laser (via rotating the grating) over the desired wavelength range in 10 s. To acquire absorbance from the measured acetone profile, the measured acetone spectrum is normalized to the background N_2_ spectrum. The estimated spectral resolution is ~1 cm^−1^ (30 GHz) which depends on the speed of the grating rotation (11 cm^−1^/s) and number of the acquired data points (~100). The measured absorption spectrum is compared to the calculated acetone spectrum using the PNNL 2014 database (for 50 ppmv of acetone with 76 m optical path length at room temperature and pressure of 100 mbar) and the residual is shown in the lower panel of Figure 5. In order to achieve a satisfactory frequency matching between the simulation and measurement, we fit a ninth order polynomial to the frequency calibration data points retrieved from the FTIR and use the retrieved function to convert the scan-time scale to the wavenumber scale. The remaining residual is mainly due to the spectral instability and deviation from the frequency calibration in each individual scan. These effects are caused by low-frequency mechanical vibrations in the laser cavity, longitudinal mode-hops due to the change in the laser cavity length, the hysteresis of the optomechanical tuning and potentially by the remaining unsuppressed higher order modes of the EC-QCL. In order to minimize the effect of the optomechanical hysteresis in frequency calibration, we used two different calibration routines for forward and backward sweeps.

**Figure 6:**
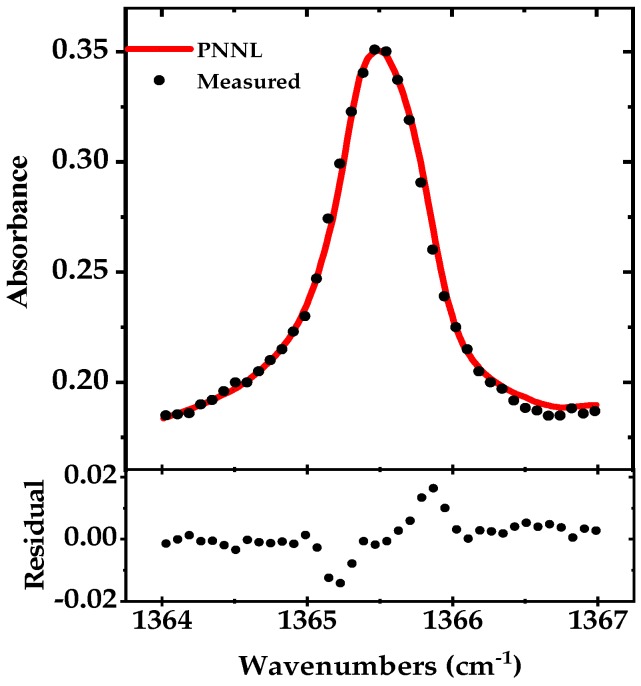
Narrowband absorption spectrum of the acetone Q-branch (~1365.5 cm^−1^) in black (100 mbar pressure, optical path length of 76 m), measured in 40 ms and averaged for five times compared to simulated absorption spectrum from the PNNL 2014 database in red. The residual is shown in the lower panel.

To record only the Q-branch absorption peak of acetone (Γ ~ 10 GHz), we rotate the grating by the piezo-actuator (mounted on top of the DC motor) at 25 Hz. Like broadband DAS, here we also used a mechanical chopper and a lock-in amplifier for detection (integration time of 1 ms), and the entire peak is scanned in less than 40 ms. Around 40 data points are recorded with a sampling rate of 1 kS/s, yielding a spectral point spacing of ~0.079 cm^−1^ (~2.4 GHz). The spectrum is averaged for five times and normalized to the background spectrum. The measured absorption spectrum is shown in Figure 6 together with a fit of the simulated absorption spectrum using the PNNL 2014 database. The residual of the fit is shown in the lower panel and the retrieved concentration from the fit is 50.0 ppmv.

**Figure 7:**
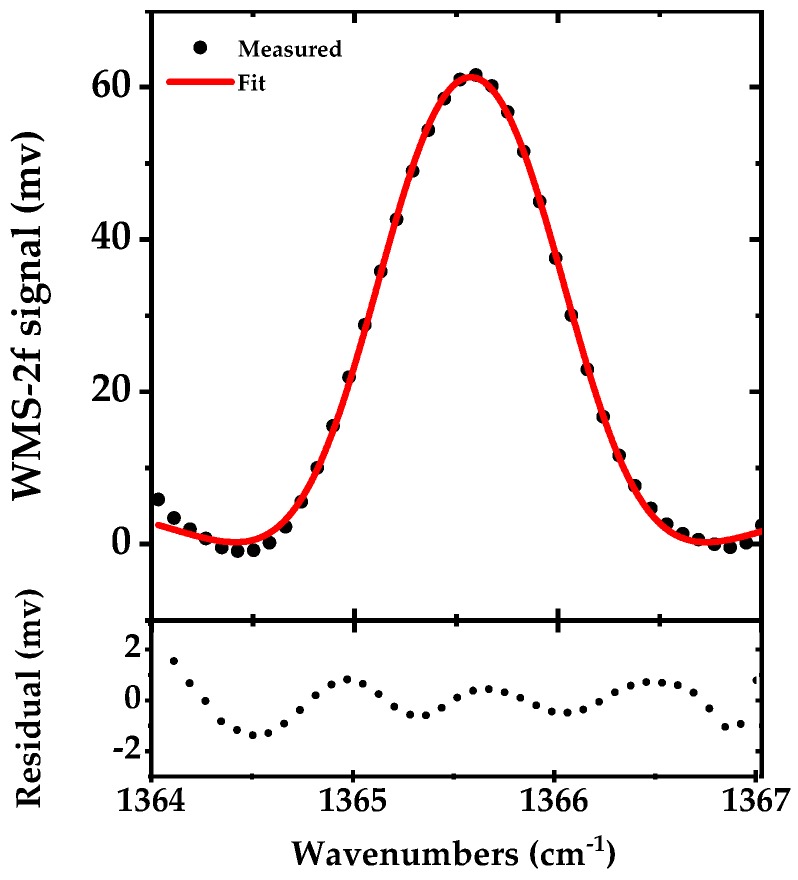
The WMS-2f signal of the acetone Q-branch (~1365.5 cm^−1^) in black (measured in 40 ms and averaged for five times), from 50 ppmv of acetone in N_2_ at 100 mbar pressure along with a fit of a simulated WMS-2f signal in red. The lower panel shows the difference between the measurement and simulation.

In DAS, the SNR is mainly limited by the 1/f noise of the system. To reduce this noise, we used scanned WMS-2f for detection of the Q-branch peak. A 50 kHz sine wave current modulation with 225 mA amplitude (in addition to a DC injection current of ~775 mA) is applied to the laser to provide the modulation amplitude of 10 GHz close to the HWHM of the Q-branch peak of acetone. To scan this modulation, the piezo of the grating is swept by a 25 Hz saw-tooth waveform and ~40 data points are recorded at a sampling rate of 1 kS/s yielding a spectral point spacing of 0.077 cm^−1^ (~2.3 GHz). The signal is demodulated by the lock-in amplifier and recorded in 40 ms. Figure 7 shows the measured signal after background correction in black along with a fit WMS-2f simulated signal in red. The residual of the fit is shown in the lower panel. The retrieved concentration from the fit is 49.1 ppmv.

The Q-branch peak of the acetone consists of multiple congested lines. Here, we used a Pseudo Voigt function to fit the WMS-2f spectra. We observed some artifacts in the residual due to lacking a proper fitting model, which most probably can be eliminated by using a proper line shape model. However, with the present fit to the measurement data, we observed an enhancement in SNR as compared to the narrowband DAS.

**Figure 8:**
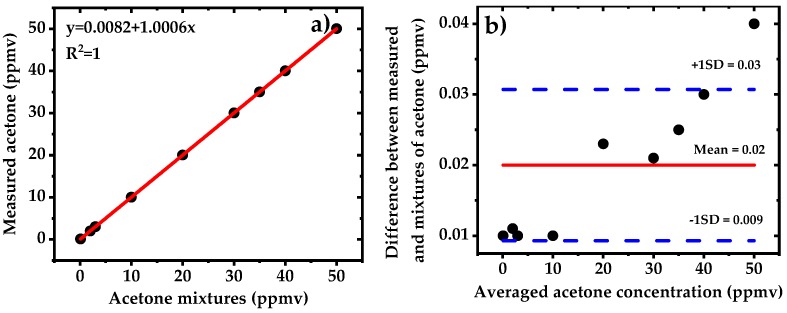
(**a**) Linearity of the WMS-2f setup. Measured acetone concentrations against the prepared dilutions of the calibrated mixtures of 50 ppmv in N_2_; (**b**) Bland–Altman plot showing the residual for 9 acetone mixtures. The solid red line represents the mean difference between the measured and applied acetone mixtures while blue dashed lines indicate the standard deviations.

The linearity of the system is characterized by measuring nine different mixtures of acetone using WMS-2f detection method. The acetone mixtures are prepared by dynamic mixing of pure N_2_ with standard calibrated bottle of 50 ppmv acetone in N_2_ down to 0.1 ppmv (100 ppbv) using two mass flow controllers. The results are displayed in Figure 8. The sensor shows a good linear response. The fit to the measured concentration yields an offset of 8.2 ppbv and a slope of 1.0006. We did not observe neither absorption saturation effect as the optical path length of 76 m yields absorbance of 0.35 for 50 ppmv of acetone nor any particular trend in the residuals. The mean value of the residuals are shown with a red line accompanied by two blue dashed lines showing the standard deviation. The relative inaccuracy of the sensor for different concentrations (mean value ~20 ppbv) is approximately within the detection limit of the signals (~15 ppbv).

The time response of the sensor is limited by the volume of the multipass cell and the gas flow through the cell which is not a bottle-neck in the system. This can be improved to sub-second timescale by using a cell with less volume, increasing the gas flow and decreasing the gas pressure in the cell (see e.g., [4]).

To measure the long-term stability of the system, we recorded a normalized reference spectrum (acetone spectrum divided by a background spectrum using pure N_2_) from the calibrated bottle of acetone. Afterward, we measured around ten thousand consecutive normalized spectra (all normalized to the same background spectrum) under the same concentration and conditions. We compared them with the reference spectrum in a linear way, i.e., the intensity of each measured absorption spectrum was plotted with the intensity of the reference absorption spectrum in *x*-*y* coordinates for all of the data points. A line was fitted to the data-set whose slope determines the acetone concentration in each individual measurement. These concentrations were recorded continuously and used to calculate the Allan deviation for evaluating the long-term stability of the system. This procedure has been already used for broadband and narrowband DAS and also WMS-2f signals [45].

**Figure 9:**
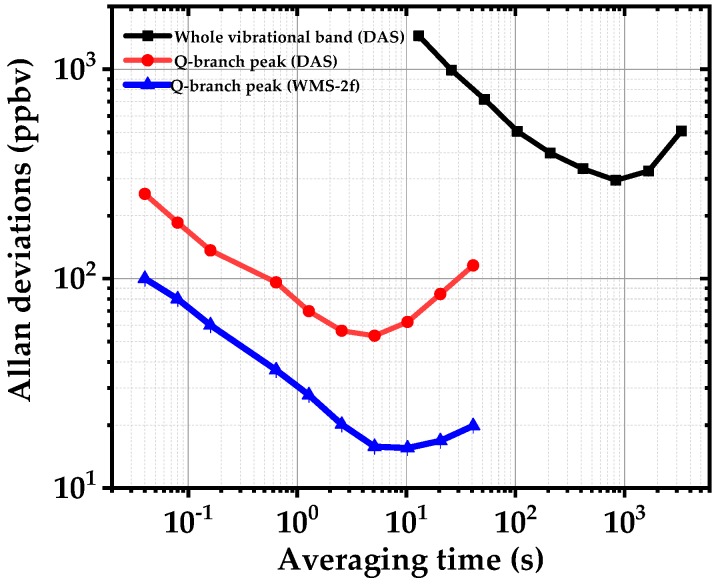
Allan–Werle deviations of the minimum detectable concentrations using scanned DAS of the complete vibrational band of acetone (1300–1420 cm^−1^) in black, DAS of the Q-branch peak near 1365 cm^−1^ in red and WMS-2f of the same peak in blue.

To evaluate the detection limit, stability and precision of the spectrometer for acetone detection, we calculate the Allan–Werle deviation for scanned DAS and WMS-2f, as shown in Figure 9. The noise-equivalent absorption sensitivity (NEAS) per measurement data point of our EC-QCL is calculated for each method by [26],

(3) $$\mathrm{NEAS}={\left(\frac{\Delta I}{I_{0}}\right)}_{min}\frac{1}{L_{eff}}\sqrt{\frac{nT}{N_{p}}},$$

in which ${\left(\Delta I/I_{0}\right)}_{min}$ is the intensity noise on the baseline without any absorber, *L_eff_* is the effective interaction length, *N_p_* is the number of the measured data points, *n* is the number of the scans, and *T* is corresponding time for a single measurement. For broadband DAS, a minimum detectable absorption (MDA) of 300 ppbv in 850 s and NEAS of 3.8 × 10^−6^ cm^−1^ Hz^−1/2^ ($\Delta I/I_{0}=4\times 10^{-2},$ *n* = 5, *T* = 10 s, *N_P_* = 100, *L_eff_* = 7600 cm) are achieved. Using a narrow scan around the Q-branch absorption peak of acetone the NEAS is improved to 9.3 × 10^−8^ cm^−1^ Hz^−1/2^ ($\Delta I/I_{0}=10^{-2}$, *n* = 5, *T* = 0.04 s, *N_P_* = 40, *L_eff_* = 7600 cm) due to a shorter acquisition time and higher SNR compared to the broadband DAS. In WMS-2f, the SNR and MDA are further improved, although we apply a modulation amplitude of ~10 GHz which is lower than the optimum modulation amplitude (22 GHz) to maximize the WMS-2f signal from the Q-branch peak of the acetone. We achieve a NEAS of 1.9 × 10^−8^ cm^−1^ Hz^−1/2^ ($\Delta I/I_{0}=2\times 10^{-3},$ *n* = 5, *T* = 0.04 s, *N_P_* = 40, *L_eff_* = 7600 cm) and a MDA of ~15 ppbv in less than 10 s. Compared to broadband DAS, a sensitivity enhancement factor of ~30 and ~100 (10 s averaging time) are achieved using narrow DAS and WMS-2f, respectively.

A comparison of the NEAS, spectral coverage and acquisition speed of all three detection schemes is shown in Table 2. Furthermore, we also compared the previously reported results of spectrometers detecting acetone around 8 µm using EC-QCL.

Broadband DAS utilizes the full gain-bandwidth of the EC-QCL laser which makes it interesting for multispecies trace gas detection of larger molecules with broadband spectral shapes, as shown by measuring the entire vibrational band of acetone from 1300–1410 cm^−1^. However, the main drawbacks of fast broadband scanning are long acquisition time, low spectral resolution and power fluctuations. The latter is due to the optomechanical tuning which causes the laser to operate in a fast longitudinal mode-hopping regime. To improve the spectral resolution and acquisition time, we switched the EC-QCL operation from broadband to narrowband DAS and targeted the Q-branch peak of the acetone. Narrowband DAS is suitable for fast detection of narrow spectral features (single absorption lines) within the scanning range of the EC-QCL. In addition, we applied a fast current modulation to the EC-QCL and targeted the acetone Q-branch peak using the WMS-2f method. WMS-2f, as shown in Figure 7 (Allan deviation in Figure 9 and Table 2), enhanced the detection limit of the acetone as compared to narrowband DAS. Its detection limit is sufficiently low to monitor the acetone levels in human exhaled breath [46,47]. Note that all of these three spectroscopy options are available with the same setup without any physical change in the source or other parts of the spectrometer. In comparison to OA-ICOS with ~1000 m effective interaction length [15,38], we obtain a similar sensitivity with WMS-2f and a multipass cell. In addition, the measurement time of the current WMS-2f system is substantially faster than OA-ICOS system. In comparison to [48] where a pulsed QCLs was used for DAS with a 54-m multipass cell, we obtain less sensitivity for the DAS operation but cover more than twice of spectrum that each pulsed QCL could cover and for WMS-2f we achieved a factor of 15 better sensitivity. The key point in comparing the two systems is the short acquisition time of the WMS-2f, which potentially enables online breath-cycle-resolved operation in breath analysis applications. Similarly, in contrast to cavity-enhanced absorption spectroscopy (CEAS) with ~5-times longer effective interaction length [49], we achieved a factor ~2 better sensitivity.

**Figure 10:**
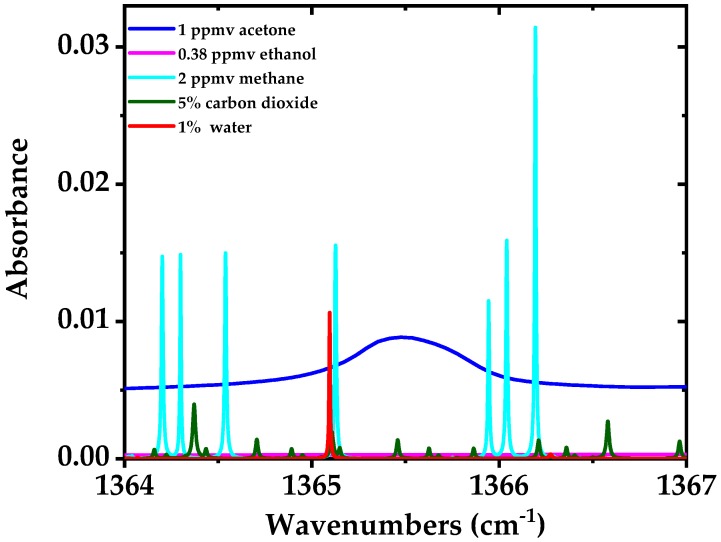
Simulated spectra of 1 ppmv of acetone (blue), 1% of H_2_O (red), 0.38 ppmv of ethanol (magenta), 5% of CO_2_ (green), and 2 ppmv of methane (cyan) in the spectral region from 1364–1367 cm^−1^ with an optical path length of 76 m, room temperature of 298 K and 100 mbar pressure [44,56].

Acetone is an important biomarker for real-time monitoring of fat burning [50] and diabetes [51]. Its concentration in breath ranges from 0.15 to 2.7 ppmv with typical values of around 0.5 ppmv [46,47], for medical diagnostics. Non-laser based methods have been successfully used for the detection of acetone in human breath; for example, chemoresistive gas sensors [52] and mass spectrometry (MS) based techniques such as proton-transfer-reaction MS and gas chromatography-MS. However, the cost of MS instruments is very high, they need sample preparation and do not offer portable operation. Laser spectroscopy provides an alternative to MS methods with lower cost, portable operation, compactness and ease of use; which has been already employed (e.g., QCLs or EC-QCLs based systems) for acetone detection in human breath or its stand-off detection [38,48,53,54]. Acetone has broadband absorption features in the mid-infrared region with a large amount of closely spaced ro-vibrational transitions. It is usually difficult to achieve the adequate sensitivity in order to measure acetone in breath using broadband absorption spectroscopy due to their limited sensitivity and/or resolution. Here we targeted the Q-branch absorption peak of acetone to achieve high spectral resolution and sensitivity in a short acquisition time. Simulated spectrum of 1 ppmv of acetone (in blue) which is a typical concentration in the breath (see e.g., [4]) in the spectral region from 1364–1367 cm^−1^ is shown in Figure 10, along with other interfering gases in this spectral region including 0.38 ppmv of ethanol (in magenta), 5% of CO_2_ (in green), 1% of H_2_O (in red), and 2 ppmv of methane (in cyan). The simulation is performed for room temperature (298 K), 100 mbar pressure, and an optical path length of 76 m. It is evident that interference of ethanol with the Q-branch peak of acetone is almost negligible. The water absorption line at 1365.1 cm^−1^ and a few methane absorption lines have interference with the Q-branch of acetone. However, these interfering species have much narrower absorption features compared to the Q-branch of acetone which makes their interfering influence less crucial for acetone measurement; it is even possible to fit them out from the measurement results if needed. Note that 1% concentration of water in the simulation, despite of higher concentration of water vapor in human breath (~6%), is due to the assumption of using water concentration reduction methods in the breath sampler, e.g., using a membrane filter with a small pore size [55].

## 7. Conclusions and Outlook

A continuous wave, single mode, widely tunable EC-QCL, with a tuning range of up to 300 cm^−1^ around 8 µm and output power of up to 20 mW, is utilized for absorption spectroscopy. The laser can be scanned over the complete tuning range in less than 30 s. The laser is combined with a 76 m long multi-pass cell to measure high-resolution broadband spectra of acetone spanning from 1300–1420 cm^−1^. Sensitive narrowband measurements of acetone spectra are performed using WMS-2f with a modulation amplitude of 10 GHz and a modulation frequency of 50 kHz on the laser current. We targeted the Q-branch peak of acetone, which has an HWHM of ~10 GHz at ~100 mbar. With WMS-2f, a minimum detection concentration of ~15 ppbv in 10 s is achieved, resulting in a NEAS of 1.9 × 10^−^^8^ cm^−1^ Hz^−^^1/2^, 2 orders of magnitude better than using broadband direct absorption spectroscopy of the complete vibrational band of acetone. Different tuning performances provide the advantage of applying a broadband scan, a narrowband scan, and also wavelength modulation without changing the light source and enable us to compare the spectral resolution, sensitivity, acquisition speed and bandwidth of the various methods. Our results show that widely tunable EC-QCLs in combination with sensitive spectroscopic methods (WMS-2f with MPC, CEAS), will allow simultaneous detection of various molecules for different applications such as breath analysis (e.g., acetone, hydrogen peroxide, hydrogen cyanide) and spoilage monitoring of agricultural and food products (e.g., ethanol, acetone, acetaldehyde, 1-3 Butadiene) [57].

## Figures and Tables

**Table 1:** Wavelength tuning performance of the EC-QCL.

Tuning	Range (cm^−1^)	Mechanism	Adv./Dis.
DC motor on the grating	300 (1120–1420)	Grating rotation	Slow, broadband
PZT on grating	~3	Grating rotation	Fast, narrowband
PZT on cavity length	~1.5	External cavity length variation	Fast, w/o intensity modulation
Current	~3	Injection current	Very fast, w/ intensity modulation, in combination with PZT mode-hop free
Thermal	~0.1–0.2 cm^−1^/K	Change of laser head temperature	Very slow

**Table 2:** Comparison of the NEAS using different detection schemes for whole vibration band of the acetone (1300–1420 cm^−1^), the narrow Q-branch peak (DAS) and its 2f modulation, along with the previously reported results of spectrometers detecting acetone around 8 µm.

	Laser Source	Detection Method	*L_eff_* (m)	Spectral Coverage (cm^−1^)	Acquisition Speed	NEAS (cm^−1^ Hz^−1/2^)
This work	EC-QCL	DAS	76	~110	10 (s)	$3.8\times 10^{-6}$
This work	EC-QCL	DAS	76	~3	40 (ms)	$9.3\times 10^{-8}$
This work	EC-QCL	WMS-2f	76	~3	40 (ms)	$1.9\times 10^{-8}$
Ref. [48]	QCL	DAS	54	100	5.3 (s)	$2.9\times 10^{-7}$
Ref. [49]	QCL	CEAS	375	~3	N/A	$1.7\times 10^{-7}$
Ref. [15]	EC-QCL	OA-ICOS	1000	~110	60 (s)	$3.7\times 10^{-8}$
